# Impact of Amorphous SiO_2_ Nanoparticles on a Living Organism: Morphological, Behavioral, and Molecular Biology Implications

**DOI:** 10.3389/fbioe.2014.00037

**Published:** 2014-09-29

**Authors:** Alfredo Ambrosone, Maria Rosaria Scotto di Vettimo, Maria Ada Malvindi, Modi Roopin, Oren Levy, Valentina Marchesano, Pier Paolo Pompa, Claudia Tortiglione, Angela Tino

**Affiliations:** ^1^Istituto di Cibernetica “Eduardo Caianiello”, Consiglio Nazionale delle Ricerche, Pozzuoli, Italy; ^2^Center for Biomolecular Nanotechnologies@UNILE, Istituto Italiano di Tecnologia, Arnesano, Italy; ^3^The Mina and Everard Goodman Faculty of Life Sciences, Bar Ilan University, Ramat Gan, Israel

**Keywords:** amorphous silica nanoparticles, *Hydra*, extracellular matrix homeostasis, GSH response, RNAseq, nanotoxicity

## Abstract

It is generally accepted that silica (SiO_2_) is not toxic. But the increasing use of silica nanoparticles (SiO_2_NPs) in many different industrial fields has prompted the careful investigation of their toxicity in biological systems. In this report, we describe the effects elicited by SiO_2_NPs on animal and cell physiology. Stable and monodisperse amorphous silica nanoparticles, 25 nM in diameter, were administered to living *Hydra vulgaris* (Cnidaria). The dose-related effects were defined by morphological and behavioral assays. The results revealed an all-or-nothing lethal toxicity with a rather high threshold (35 nM NPs) and a LT50 of 38 h. At sub lethal doses, the morphophysiological effects included: animal morphology alterations, paralysis of the gastric region, disorganization and depletion of tentacle specialized cells, increase of apoptotic and collapsed cells, and reduction of the epithelial cell proliferation rate. Transcriptome analysis (RNAseq) revealed 45 differentially expressed genes, mostly involved in stress response and cuticle renovation. Our results show that *Hydra* reacts to SiO_2_NPs, is able to rebalance the animal homeostasis up to a relatively high doses of SiO_2_NPs, and that the physiological modifications are transduced to gene expression modulation.

## Introduction

Silicon is an abundant element in the earth’s crust and biota. Starting from the prehistoric flints, humans have used materials containing natural silicon and its compound to build artifacts. Today, silica-based components are present in every digital devices that enhance the modern human activities. In the near future, nano-biotechnological approaches will deliver many nanoscale smart sensors and drugs to solve biomedical issues and there is a great expectation on silica-based nanostructures. Amorphous silica can be of natural origin (Diatoms) or synthetic (silica-gel, colloidal). Aggregates of amorphous silica of micrometer size have been used for years in oral and dermatological formulations, medicines, food, and cosmetics; hence, new nanomaterials containing SiO_2_ are considered low toxic and moderately biocompatible. However, the fast growing knowledge in the nanoscience fields has confirmed that, at nano-dimension, materials can acquire new features (Napierska et al., 2010). This is true also for the amorphous silica: in fact, if nanoparticle aggregates ranging from a few micrometers up to millimeter are rather irritant but non-toxic, on the other hand, SiO_2_ nanoparticles (SiO_2_NPs) show a dose-related toxicity in *in vitro* experiments (Napierska et al., 2010). In water, SiO_2_ surface exhibits silanol groups (Si–O–H). At nanoscale, the high density of silanol groups on SiO_2_NPs form several siloxane framework architectures making SiO_2_NPs extremely reactive and prone to be modified by environmental factors. These architectures are combinations of closed siloxane rings, along with the spatial arrangement, pattern, and degree of hydrogen bonding that terminate the siloxane rings at the silica NP surface; in addition, some scientists have also hypothesized surface-associated radicals inducing ROS species (Gazzano et al., 2012; Zhang et al., 2012).

In recent years, many new synthetic routes to produce amorphous silica stabilized nanoparticles have been developed and a few publications describe that even the synthetic procedure can influence their effects on cells (Zhang et al., 2012) and living systems, therefore, the assessment of the structure–activity relationships for amorphous silica is problematic. While crystalline silica is structurally well-defined, amorphous silica lack long-range order, and, due to a flat energy landscape, their structures are strongly dependent on synthetic procedures. Therefore, it is necessary to associate to each synthetic procedure an exhaustive description of the effects produced in living organisms: (i) to explain the mechanism of action of new materials, (ii) to open new ways on novel material application potentialities, (iii) to create a database of material/living-systems interactions.

The aim of this study is to investigate the possible toxicity of SiO_2_NPs at animal level. Malvindi et al. (2012) synthesized, through the microemulsion methodology, stable and monodisperse silica nanoparticles, and accurately characterized their physico/chemical properties. Through an *in vitro* approach, they described (within the picomolar–nanomolar range) the cellular uptake and the absence of toxic effects. Here, we describe the effects of SiO_2_NPs, synthesized following the same procedure, on a small model organism, the coelenterate *Hydra vulgaris*.

*Hydra* is a freshwater invertebrate; organized in two epithelia (ectoderm, endoderm) whose cells originate from three cell lineages that differentiate in more than 15 types of specialized cells (armed, secretory, digestive, nervous, myoepithelial) (Hobmayer et al., 1990). Cell type composition follows defined proportions and can be analyzed by macerating the animals in fixed cell suspensions (David, 1973). *Hydra* anatomy can be summarized as a hollow tube with a tentacles crown. As for many other animals, *Hydra* is covered by a cuticle, an extracellular matrix secreted by ectodermal cells that forms the distal interface with the environment. It has been shown that *Hydra* cuticle proteins from the PPOD and SWT families are associated with chondroitin and chondroitin 6-sulfate (Bottger et al., 2012). The behavior of *Hydra* is well characterized; the gastric region expands and contracts spontaneously with a defined pace (Ruggieri et al., 2004). *Hydra* undertakes active feeding, and this behavior that can be mimicked by Glutathione (GSH) (Lenhoff, 1961; Westfall and Kinnamon, 1984). These two peculiar aspects of *Hydra* biology are considered in this work in the context of interaction with new nanomaterials; traditionally used in developmental, regenerative, and evolutionary research contexts (Galliot et al., 2009; Bode, 2011) *Hydra* is actually a good biological indicator for environmental studies (Karntanut and Pascoe, 2002). In addition, stress response genes are conserved and at least in part characterized (Martinez and Bridge, 2012). In previous works, we have successfully exploited the potential of *Hydra* to describe the mechanisms of action underlying the interaction of nanoparticles with living organisms and the animal toxicity of new materials establishing reliable methods to evaluate at multiple levels their toxic effect (Tortiglione et al., 2007, 2009; Malvindi et al., 2008; Ambrosone et al., 2012; Ambrosone and Tortiglione, 2013; Marchesano et al., 2013).

Here, we determined a wide array of toxicity endpoints caused by SiO_2_NPs, spanning from behavior, morphology, population growth rate, cell proliferation/apoptosis balance up to gene expression profiling. *Hydra* transcriptomic changes further suggested that even in absence of severe alterations, treatment with SiO_2_NPs causes effects that can be transduced to the nucleus and influence gene expression to ultimately modify principal cell functions such as cuticle renovation and response to stress.

## Materials and Methods

### Synthesis of SiO_2_NPs in a ternary w/o microemulsion (25 nm)

SiO_2_NPs were prepared by following recently optimized synthesis procedure (Malvindi et al., 2012). The ternary microemulsion synthesis permits to obtain 25 nM SiO_2_NPs highly monodispersed and stable. The ultrasonic treatment was used to completely disperse the SiO_2_NPs before the treatments. The surface of the nanoparticles was modified with amine groups using 5% (v/v) solution of aminopropyltrietoxysilane (APTES, Sigma Aldrich) and 1 mM acetic acid (99.7%, Sigma Aldrich) and stirring for 60 min. Fluorescent SiO_2_NPs were prepared adding to the microemulsion 50 μL of Rhodamine isothiocyanate (TRITC) dye, previously modified as above described, so TRITC was covalently linked inside SiO_2_NPs (Guarnieri et al., 2014).

### Culture of test organisms

*Hydra vulgaris* were cultured in *Hydra* medium consisting of 1 mM calcium chloride, 0.1 mM sodium hydrogen carbonate, pH 7 (Loomis and Lenhoff, 1956). Animals were fed on alternate days with *Artemia nauplii* at 18°C with a 12:12 hours light:dark regime. Polyps from homogeneous populations, 3-weeks-old and carrying one or two buds, were selected for the experiments.

### Morphological and behavioral assays

#### Toxicity tests

Toxicity tests were carried out on groups of 25 polyps for 24, 44, 48, 50, 72 h at SiO_2_NP concentrations of 10, 15, 25, 35, 50 nM. Either control or treated animals were placed into plastic multiwells refreshing the medium every 24 h. The morphophysiological effects were recorded by microscopic examination of each polyp and used to extrapolate the score key reported in Table 1, ranging from 7 for a normal polyp to 1 for polyps irreversibly damaged to death. The median scores were analyzed at 24, 44, and 50 h for each concentration of SiO_2_NPs. On the base of these outcomes, lethal dose and lethal time were calculated by applying the trimmed Spearman–Karber method (Hamilton et al., 1977).

**Table 1:** **Description of morphological traits and vital states of SiO_2_NPs treated *Hydra* and corresponding score key**.

Score	Morphology	Vital state
7	Normal hydra	Active
6	Bumped gastric region, normal tentacles	Active
5	Inverted-cone gastric region; shortened tentacles	Whitening, gastric region paralyzed, slow tentacles
4	Inverted-cone gastric region, short tentacles	Paralyzed, rigid tentacles
3	Gastric region reduced in size, crushed tentacles	Permanently damaged to death
2	Further reduced gastric region, without tentacles	Abundant cell loss
1	Irregular shape	Death
0	Dispersed cell clumps and debris	Disintegrated

#### Tentacle structure and battery cell complex organization

Tentacle structure and battery cell complex organization were evaluated by toluidine-blue staining. Animals were relaxed for 1 min in a drop of 2% urethane in culture solution and fixed by pouring 99.5% ethanol. Five minutes later, the animals were rinsed several times in distilled water to remove the ethanol and then stained with 0.05% toluidine blue in 10 mM Tris–HCl (pH 7.5). The animals were rinsed several times to remove an excess of dye and dehydrated stepwise with 50, 75, and 95% ethanol and twice in 100% ethanol. The animals were cleared in xylene and mounted in DPX, a non-aqueous mounting medium (Fujisawa, 1992). Stained tentacles were observed under bright field and phase-contrast microscopy (Axiovert 100, Zeiss) equipped with a digital color camera (Olympus, DP70). Morphological observation and quantitative analysis of isolated battery cell complexes was carried out on DAPI counterstained macerates obtained as described below.

#### Feeding assay

Fifteen polyps either showing the inverted-cone (score 5) morphophysiology or untreated, were fed with living *artemia* or treated with GSH (10 μM). The feeding behavior and the GSH response were examined under a stereomicroscope equipped with a CCD camera (Olympus).

### Macerates, BrdU assay, and assessment of apoptosis

#### Macerates

*Hydra* tissues were dissociated in a modified maceration solution containing glycerol: acetic acid: water (1:1:7) at room temperature. Identification of cell types was also done according to the classification given by David (1973). Through this method, every cell type can be easily identified and investigated.

#### BrdU assay

To estimate the cell proliferation rate, intact *Hydra*, untreated or treated with 25 nM SiO_2_NPs, were continuously incubated with 5 nM bromodeoxyuridine (BrdU) (Sigma) for 12, 24, 48 h. Animals were macerated as described above, immediately fixed with 4% paraformaldehyde, and the cells spread on microscope slides. Incorporation of BrdU in proliferating cells was detected by immunolocalization using mouse anti BrdU monoclonal antibody (1:500, Sigma), and Novolinker polymer detection system (Novocastra) according to the manufacturer’s instructions. Epithelial and interstitial BrdU-labeled cells were observed and counted under bright field and phase-contrast microscopy (Axiovert 100, Zeiss) equipped with a digital color camera (Olympus, DP70).

#### Apoptosis

As apoptotic hallmark, pyknotic nuclei were evaluated by DAPI (4′-6-diamidino-2-phenylindole) staining. Briefly, untreated and SiO_2_NPs treated polyps (24 h; 10, 25 nM) were macerated as above described. After extensive washing in PBS, macerates were stained with DAPI for 2 min and washed in PBS. Slides were observed with phase-contrast and fluorescent microscopy to detect pyknotic nuclei. Three hundred epithelial cells were counted for each treatment and the percentage of apoptotic nuclei was determined.

### Estimation of the Silicon content by optical microscopy and by ICP-AES

Living *Hydra* exposed to Rhodamine-labeled SiO_2_NPs (25 nM) were observed under fluorescent microscopy after 24 h of treatment.

To estimate the intracellular Silicon, replicates of 150 *Hydra* were either exposed to 10 and 25 nM SiO_2_NPs (experiment) or maintained in culture solution (control) for 24 h. The incubations were carried out in 10 mL. Samples were washed and digested by the addition of HCl/HNO_3_ 3:1 (v/v) solution. The incubation media were recovered and digested as for samples. The resulting solutions were directly analyzed to evaluate the Si concentration through elemental analysis that was carried out by inductively coupled plasma atomic emission spectroscopy (ICP-AES) with a Varian Vista AX spectrometer.

### *Hydra* growth rates and regeneration

#### Growth curve

Groups of five *Hydra* (with one bud) were treated with 10, 25 nM of SiO_2_NPs for 24 h or untreated, washed, and the following day placed in 12-well plates (1 *Hydra*/well). *Hydra* were fed once daily for 18 days. The growth rate constants (*k*) for the three populations were determined, as previously described (Ambrosone et al., 2012), from ln(*n*/*n*_0_) = *k*_t_, where *n* is the number of animals at time *t* and *n_0_* the number of animal at *t*_0_. For *n/n*_0_ = 2, *t* = *T*_2_, the doubling time of the population. *T*_2_ was determined by linear regression (Bosch and David, 1984).

#### Regeneration assay

Regeneration assay groups of 25 polyps were bisected in the upper gastric region and incubated in presence of 10 and 25 nM SiO_2_NPs. The regenerating polyps, monitored through a stereomicroscope, were grouped into three stages according to progressive tentacle morphogenesis (Ambrosone et al., 2012).

### RNA extraction and qRT-PCR

Total RNA from treated and untreated animals was extracted using Trizol (Life Technologies) and further purified by silica-cartridge purification protocol from the PureLink RNA Mini Kit (Ambion). RNA concentration was determined on the NanoDrop ND-1000 spectrophotometer (Thermo Scientific, USA). The first-strand cDNA synthesis was carried out by High-Capacity cDNA Reverse Transcription Kit (Applied Biosystems), using 0.5 μg of DNA-free RNA in a final volume of 25 μl according to the manufacturer’s instructions. Real-time RT-PCR (qRT-PCR) was performed in 25 μl of reaction mixture consisting of 1× Express Sybr^®^ GreenER qPCR SuperMix with premixed ROX (Invitrogen), serial cDNA dilutions, and 0.3 μM of each primer. The reactions were processed using the Step One Real-Time PCR System (Applied Biosystem) under the following fast cycling steps: initial denaturation for 2 min at 94°C, followed by 40 cycles at 94°C for 2 s and 59°C for 30 s. In addition, melting curves (20 min; from 59 to 90°C) were generated to check any spurious amplification products. To normalize RNA levels, *Hydra* Elongation factor 1α gene (HyEf-1α, GenBank Accession no. Z68181.1) was employed as internal calibrator. Nucleotide sequences and alignments were obtained from *Hydra* genome database^1^ and European Nucleotide Archive (ENA)^2^. Specific primers of *Hydra* homologs genes were designed using Primer3 software^3^ and are listed in the Table S1 in Supplementary Material). Gene expression was evaluated in SiO_2_NPs treated and untreated polyps; at least three technical repeats from three biological replicates were carried out. Herein, the delta–delta Ct (2^−ΔΔCT^) method, for comparing relative expression results between treatments, was applied (Livak and Schmittgen, 2001).

### RNA-seq analysis

Prior to sequencing, RNA amount and quality were quantified by capillary electrophoresis on a microchip device (BioAnalyzer 2100, Agilent). Samples with the RNA integrity number (RIN) of at least nine were chosen for further analysis. RNA sequencing was carried out at the Institute of Applied Genomics (IGA, Udine, Italy) on an Illumina Genome Analyzer, Hi-Seq2000, performing 100 bp-paired end sequencing in a single lane according to the manufacturer’s instructions. The sequenced reads resulting from this run were separated into 12 libraries (representing three related but different projects) according to their indexes (de-multiplexing) and stored as FASTQ files. The SiO_2_NPs treated and control *Hydra* libraries yielded a total of 30 million reads. Specifically, these libraries included (in millions of reads): 13.5 and 16.5 for SiO_2_NPs treatment and control, respectively.

Raw RNA-Seq reads were trimmed according to base calling quality prior to analysis. Quality filtering was performed using FastQC^4^. Based on quality statistics, RNA-Seq reads were not trimmed (Phred scores >Q25). RNA-Seq reads were mapped to the reference using the BWA alignment program (Li and Durbin, 2009) according to default parameters^5^. A well annotated *Hydra* transcriptome, which has recently been deposited at the ENA under the project number PRJEB445 and with accession numbers from HAAC01000001–HAAC01045269 (Wenger and Galliot, 2013) was used as reference to calculate transcript coverage. Specifically, the coverage of each transcript (i.e., the number of reads mapped to it) was computed using BEDtools software. Counting the number of aligned reads per transcript per sample was performed BEDTools “multicov” tool with the −D flag, thus enabling counting of reads that were mapped to more than one transcript with the same primary alignment score. Raw read counts and normalized counts were extracted from files using the multicov option of BEDtools (Quinlan and Hall, 2010). The count data produced from mapped reads were then processed with the DESeq R package (Anders and Huber, 2010), which tests for differential expression based on the negative binomial distribution while normalizing the raw counts to account for variable library sizes. Differentially expressed (DE) genes were identified through comparison of a SiO_2_NP-treated sample to an untreated control as reference group. Comparisons were kept at a significance level cut-off of α = 0.05 adjusted to match a 20% false discovery rate using the Benjamini–Hochberg procedure. An additional minimum threshold of fold change of two was also used. A consensus list of DE genes was generated from the latter. The Blast2GO software suite (Gotz et al., 2008) was then used to predict transcript function and assign Gene Ontology (GO) terms (Ashburner et al., 2000; Gene Ontology, 2008) for significantly up and down regulated transcripts without annotation. Blast2GO parameters included BLASTX against the GenBank non-redundant (nr) database hosted by the National Center for Biotechnology Information (NCBI)^6^ while applying a minimum *E*-value cut-off score of 1.0E−06 and retrieving a maximum of 30 blast hits for each transcript. GO functional terms and Interpro domain annotations to each of the mapped un-annotated sequences were conducted using the Blast2GO default parameter settings.

## Results

### Determination of toxicity endpoints

**Figure 1:**
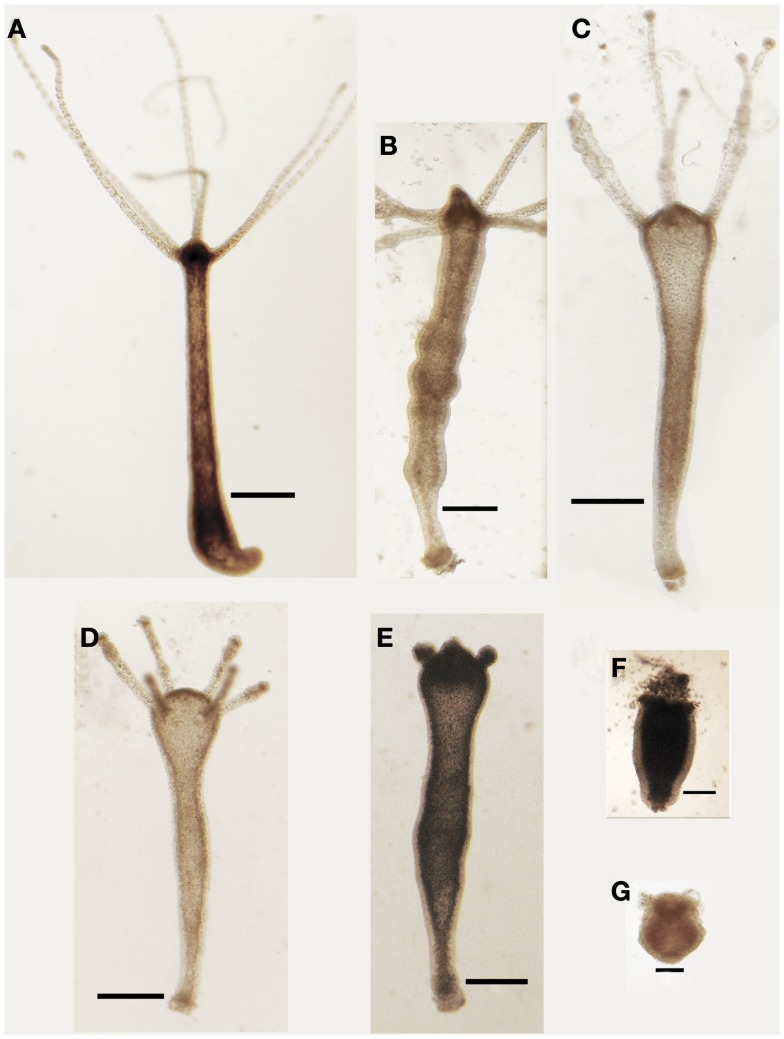
**Effects SiO_2_NPs treatment on *Hydra* morphology**. Representative *in vivo* images of major features of morphological alterations (see Table 1) induced by continuous exposure to SiO_2_NPs are reported into the figures. Bar in **(A–E)**, 1 mm; in **(F,G)**, 0.5 mm.

Living animals were soaked in culture medium supplemented with increasing SiO_2_NPs doses (from 10 to 50 nM) and monitored from 24 to 72 h of incubation; progressive morphological damages were observed, as shown by representative images of Figure 1. The treatment induced an inverted-cone shaped body, whitening, and paralysis, followed by whole polyp disintegration and death.

We classified these new features using a new score key (Table 1) ranging from 7 (indicative of healthy condition) to 1 (damaged to death). In Table 1, the morphological, behavioral, and vital-states indicators toward the matching scores are described in detail.

**Figure 2:**
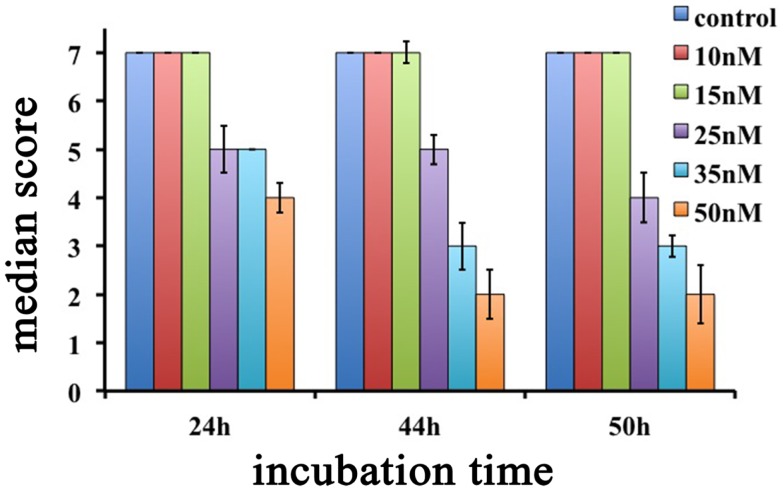
**Dose response effects of SiO_2_NPs treatments on *Hydra* morphology are shown**. Median scores were obtained from groups of 20 *Hydra* incubated for 24, 44, and 50 h with increasing concentrations of SiO_2_NPs from 10 to 50 nM.

Interestingly, *Hydra* exhibiting scores 6, 5, and 4 were found able to revert to normal upon SiO_2_NPs removal, while those showing score 3, 2, and 1 were irreversibly damaged to death. SiO_2_NPs functionalized with NH_2_ groups were also tested within the same concentration range. These NPs, even at highest concentration, did not induce recognizable effects, paralleling with data obtained from cell culture experiments (Malvindi et al., 2012). According to the key described above, the dose and incubation time effects of SiO_2_NPs were analyzed (Figure 2). Up to reasonably high concentration (15 nM), even after prolonged exposure (72 h), no toxicity was detected (data not shown). By contrast, at 25 nM *Hydra* morphology and contractility were affected though able to revert to normal condition upon SiO_2_NPs withdrawal even after prolonged incubation times (72 h) (not shown). SiO_2_NPs doses above 35 nM severely affected *Hydra* morphology causing irreversible damages after prolonged incubation (above 40 h) up to animal death. In order to determine the lethal condition, morphological scores were further analyzed. The dose response experiments showed an all-or-nothing effect with a threshold of 35 nM SiO_2_NPs at 44 h: above this concentration, the scores drop below 3 (indicating animal death) while at lower concentrations and/or shorter incubation times, the polyps never reach the lethality. At the threshold condition (35 nM), we calculated the LT50 by applying the trimmed Spearman–Karber method, which offers good statistics, is easy to use, and is recommended for accurate and precise calculation of LT50 values and their 95% confidence interval end points (Hamilton et al., 1977). The estimated LT50 was 38 h (lower confidence 33 h upper confidence 44 h) clearly indicating a toxic effect around this temporal window.

Overall, the two different methods, based on morphophysiological changes and on lethality, enabled us to determinate sub-lethal doses to assess SiO_2_NPs sub-acute effects.

### SiO_2_NPs affect distribution but not functionality of tentacle cells

In order to test whether anatomical and contractility modifications induced by SiO_2_NPs impaired also feeding behavior, we analyzed the ability of treated *Hydra* to catch the prey and respond to GSH. The feeding behavior starts with the prey capture by cnidocytes-armed tentacles followed by mouth opening and insertion of the prey-loaded tentacle into the mouth (Movie M1 in Supplementary Material). This multistep behavior can be mimicked by the tripeptide GSH. Within the 1–10 μM range, *Hydra* undergoes the GSH-response: during first step (10 s), a characteristic tentacles-writhing toward the central vertical axis of the animal takes place, followed by the second step when the tentacles bend toward the mouth culminating in mouth opening (30 s) (Movie M3 in Supplementary Material) (Lenhoff, 1961; Pierobon et al., 2001, 2004; Tortiglione et al., 2007). Treated *Hydra* (25 nM, 24 h) were challenged either with food (swimming *Artemia salina nauplii*) or GSH. In the presence of living *nauplii*, polyps thought paralyzed at gastric region and presenting the inverted-cone morphology, were still able to capture the prey; nevertheless, tentacles could not bend and mouth opening was prevented (Movie M2 in Supplementary Material). Similarly, the GSH response was impaired showing a slow tentacle-writhing activity, tentacle rigidity, and inhibition of mouth opening (Movie M4 in Supplementary Material). These results besides showing the feeding behavior impairment, revealed through the untangling of the prey capture from mouth opening that, as previously suggested by *in vitro* experiments (Thurm et al., 2004), the nematocysts discharge (the ability to catch the prey) is not sufficient to induce mouth opening. In addition, here we show that the ability of gastric region to contract must have a role in the accomplishment of feeding behavior. Since battery cell complexes are considered as the cellular correlates to food recognition, we analyzed the morphology of these cells in treated polyps by toluidine-blue staining and visualization of their nematocytes content and spatial distribution.

**Figure 3:**
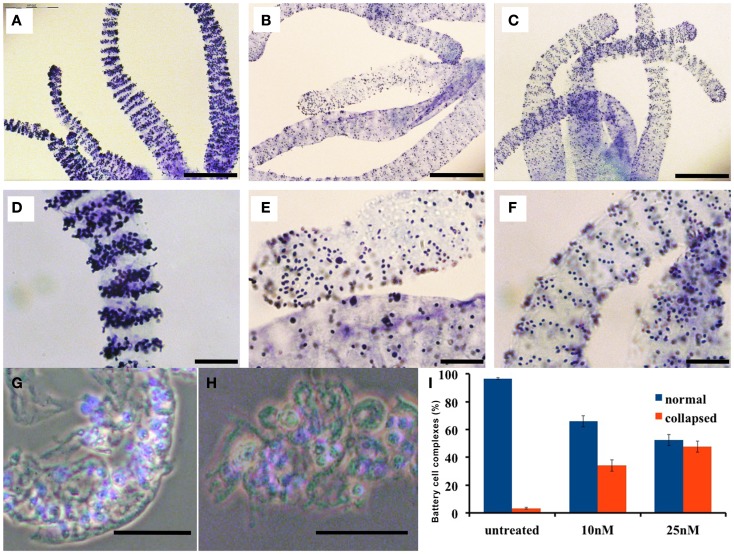
**Effects of SiO_2_NPs on tentacle structure are shown**. Nematocytes, packed into tentacle battery cell complexes, were stained with toluidine blue. Battery cell complexes distribution in normal tentacles **(A,D)**. Distribution of battery cell complexes in animals treated 24 h with 25 nM SiO_2_NPs **(B,E)**. The abnormal distribution of the complexes is recovered within 48 h from stimulus removal as shown in **(C,F)**. Effects of SiO_2_NPs on battery cell morphology cell. Isolated normal battery cell complex **(G)**, battery cell complex from treated animal **(H)**. **(I)** Quantitative estimation of the aberrant battery cells. The graph represents the percentage of collapsed battery cell complexes (red bar) at 10 and 25 nM SiO_2_NPs after 24 h incubation compared to control relative to a sample of 100 battery cells complexes; data are means of three independent experiments; vertical bars indicate the standard deviation. In **(G–I)**, either treated and control animals were macerated and the nuclei were counterstained with DAPI. Bar in **(A–C)** 200 μm; **(D–H)** 50 μm.

In normal *Hydra*, Toluidine-blue contrast highlights transversal punctuated stripes around the tentacles (Figures 3A,D), treated animals (inverted-cone stage corresponding to score 5) revealed a severe depletion of nematocytes, and a disorganization of the battery cells throughout the tentacles see Figures 3B,E. On *Hydra* macerates, the damaged battery cells lose the crescent-shaped morphology resulting shrunken, rounded, and reduced in dimension (see Figures 3G,H). In order to quantify the damage entity, we counted the number of collapsed battery cell complexes. The quantitative analysis showed that after 24 h of treatment, 50% of battery cell complexes lose their typical shape (Figure 3I). Remarkably, following SiO_2_NPs removal, *Hydra* rescues the anatomical and cellular features within 48 h (Figures 3C,F) while other important impaired traits (i.e., whitening and contractility of the gastric region) were restored even faster (1 h).

### SiO_2_NPs treatments undermine *Hydra* regenerative potential

**Figure 4:**
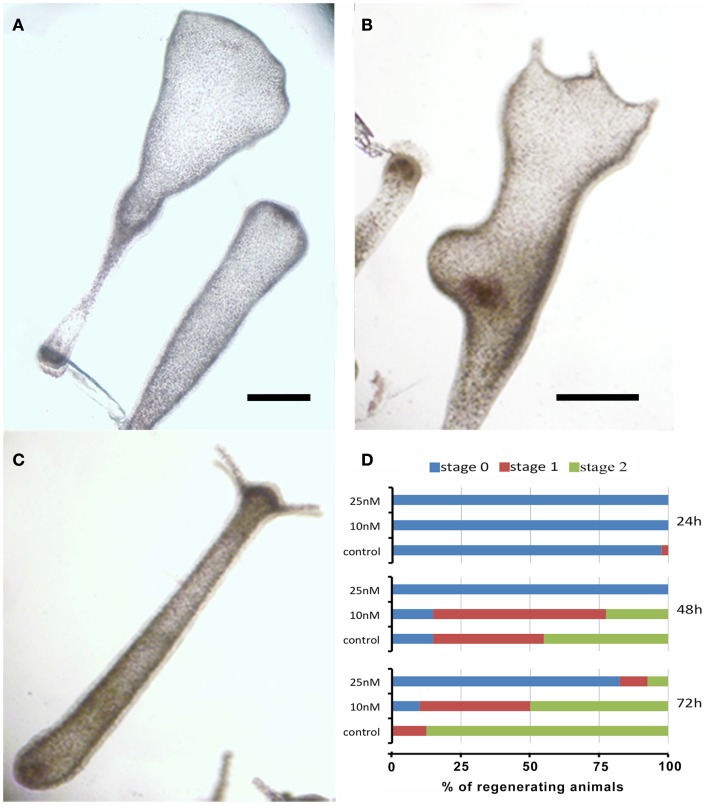
**Effects of SiO_2_NPs on *Hydra* regeneration**. Forty eight-hours-regenerating *Hydra* treated with 25 nM SiO_2_NPs **(A)**; 48 h-regenerating *Hydra* treated with 10 nM SiO_2_NPs **(B)**; untreated 48 h-regenerating *Hydra* **(C)**. In **(D)**, the histogram shows the distribution of polyps developmental stages; stage 0 indicates absence of tentacles; stage 1 indicates the presence of tentacle buds; stage 2 indicates new emerging tentacles. Animals treated with 25 nM SiO_2_NPs are significantly impaired in the regeneration process. Data are means of three independent experiments. Twenty-five animals for each experiment were employed. Bar **(A–C)**, 1 mm.

The remarkable capability of *Hydra* to regenerate missing body parts following amputation is controlled by tightly regulated processes, whose dynamics can be affected by environmental stress and profoundly altered due to failure of the differentiation, proliferation, and migration of different cell types. To test the effects of SiO_2_NPs on the polyp regeneration potential, treated adults were amputated at sub-hypostomal region and the head regeneration followed for 72 h in presence of SiO_2_NPs. The treatment severely impaired the morphology and kinetic of regeneration. Figures 4A,B show the aberrant process of treated polyps compared to control (Figure 4C); the graph in Figure 4D shows the distribution of the progressive regenerating stages: we found 50% impairment of regeneration process at 48 and 72 h post amputation (p.a.) at 10 nM, while at 25 nM, this percentage increased to 90, indicating a profound effect of SiO_2_NPs on this physiological process.

### Internalized SiO_2_NPs are confined into the ectoderm and affects the cell proliferation/apoptosis balance

**Figure 5:**
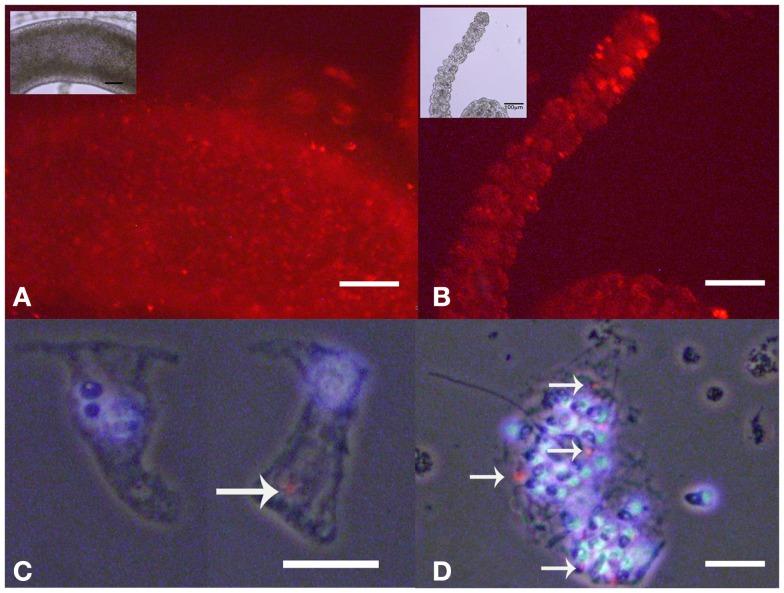
**Living *Hydra* treated with 25 nM rhodamine-labeled-SiO_2_ NPs**. Fluorescent microscopy *in vivo* images of gastric region **(A)** and tentacles **(B)**; the insets are the corresponding bright field images. Localization of SiO_2_NPs on cell maceration of *Hydra* treated as above; epitheliomuscolar cell **(C)**; battery cell complex **(D)**. Nuclei are contrasted with DAPI; SiO_2_NPs/rhodamine (arrowed red spot) localize in the apical part of epithelial cell **(C)** or tied into the nematocytes inserted into the battery cell complex **(D)**. **(C,D)** Phase contrast, DAPI, and rhodamine triple merged images. Bar in **(A,B)** 100 μm; in **(C,D)** 25 μm.

As internalized SiO_2_NPs in *Hydra* do not give any specific contrast under bright field or fluorescence microscope, imaging of the SiO_2_NPs internalization was carried out by fluorescently labeled SiO_2_NPs obtained by incorporating the dye Rhodamine B into the silica framework during the synthesis of the nanoparticles. Living *Hydra* were treated with 25 nM SiO_2_NPs labeled with rhodamine and, after 24 h, observed under fluorescent microscope. The fluorescence pattern appeared dotted and confined to the ectoderm along the tentacles and gastric region (Figures 5A,B).

The analysis of isolated macerated cells confirmed that fluorescent SiO_2_NPs localize into the cytoplasm of ectodermal epithelial cells and battery cell complexes (Figures 5C,D, respectively). To estimate the internalization rate of SiO_2_NPs, we performed inductively coupled plasma spectroscopy (ICP) measurements; replicates of 150 *Hydra* were either exposed to SiO_2_NPs (experiment) or maintained in culture solution (control) for 24 h and processed for elemental analysis. The ICP measurement confirmed Silicon presence in *Hydra*, but showed similar internalization rate at both tested SiO_2_NPs concentrations (see Table 2). This suggesting that the dose response traits described above, might concern, besides the internalized moiety, an additional fraction of SiO_2_NPs acting extracellularly.

**Table 2:** **Content of Si (element) in treated *Hydra* and incubation medium (S/N). The data are expressed as nanomoles**.

SiO_2_NPs (nM)	Hydra	Medium
10	52.3 ± 3.6	272.7 ± 28.5
25	41.7 ± 0.8	614.6 ± 17.8

**Figure 6:**
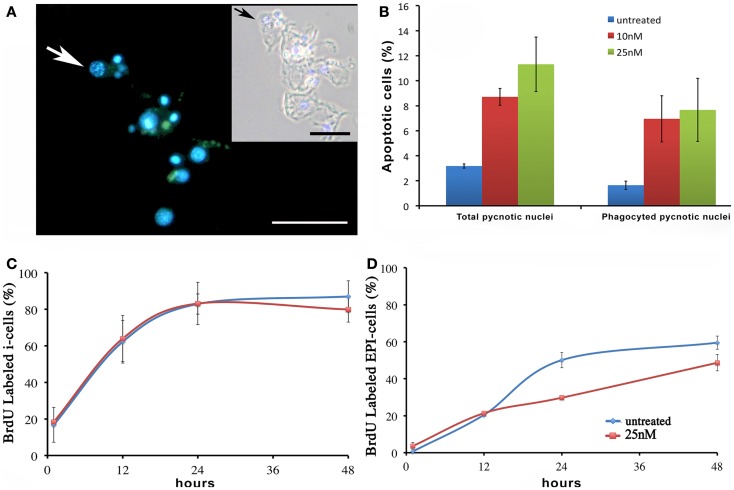
**Effect of SiO_2_NPs on cell proliferation and death**. **(A,B)** Cellular assessment of apoptosis induction by SiO_2_NPs. Following 24 h in 25 nM SiO_2_NPs, polyps were macerated and the percentage of apoptotic cells was determined by counting the DAPI-stained fragmented nuclei [arrow in **(A)**]. Scale bar: 50 μm. Inset in **(A)**: phase contrast–DAPI merged of the same image. The arrow in the inset shows a pyknotic nucleus. The graph in **(B)** shows the percentage of total pyknotic nuclei and phagocytic moiety in normal and treated conditions. Cell cycling activity of interstitial stem cells (i-cells) **(C)** and of epithelial cells (EPI) **(D)** were estimated in untreated and 25 nM SiO_2_NPs treated animals by continuous incubation with BrdU, followed by maceration of 10 animals at the indicated time points followed by colorimetric immunostaining. SiO_2_NPs impaired the proliferating activity of epithelial, as shown by the lower percentage of BrdU labeled nuclei. In **(B–D)** data represent mean ± SD of three independent experiments; 100 cells were counted for each experiment.

**Figure 7:**
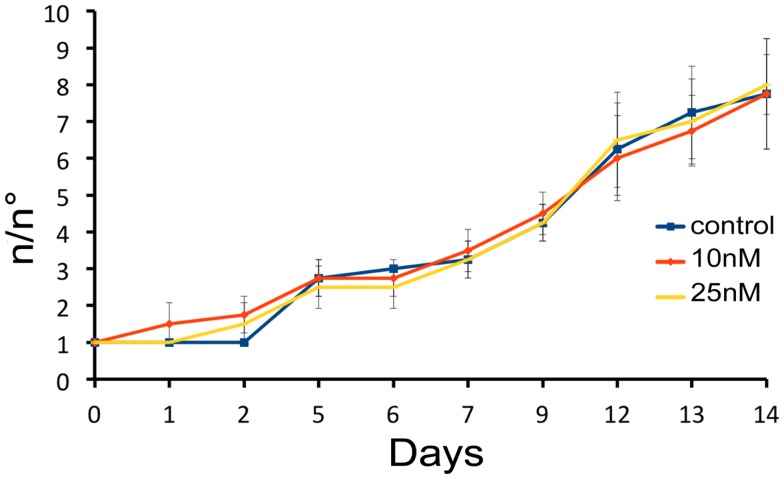
**Effect of SiO_2_NPs on *Hydra* reproduction**. *Hydra* population growth test. The graph shows *n*/*n*_0_ values at each time point. Where *n* is the total number of polyps, and *n*_0_ is the number of founder polyps.

We next investigated the influence of SiO_2_NPs treatment on cell cycle and apoptotic/proliferating balance. DAPI counterstaining on treated *Hydra* showed that the amount of phagocytosed and pyknotic nuclei was about fourfold higher compared to untreated animals (Figures 6A,B). The proliferation assay by means of BrdU labeling showed that the SiO_2_NPs treatment does not influence interstitial cell proliferation (Figure 6C) while affecting this process in epithelial cells (Figure 6D) though limited at 24 h. As the cell proliferation may affect the reproductive rate of the polyp (occurring through mitotic asexual reproduction), we assessed whether the decreased epithelial cell proliferation could influence the population growth rate. To this aim, the progeny of five treated polyps (considered as the population founders), was monitored along 2 weeks, under normal feeding regime. As shown in Figure 7, treatments with two sub lethal SiO_2_NP doses (10 and 25 nM; 24 h) did not influence the population growth rate according to T2 of 4.8 days (day 14) and *k* values corresponding to 0.50, 0.45, 0.50, respectively to control, 10 and 25 nM treatments. These data indicate that short-term SiO_2_ treatment does not affect reproductive capabilities.

### SiO_2_NPs modulate the gene expression

**Figure 8:**
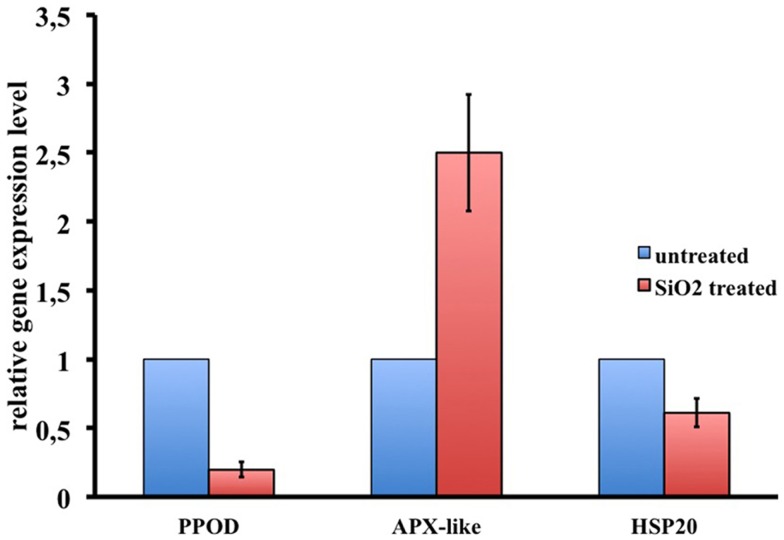
**RNAseq results validation by qRT-PCR**. Relative expression levels of three selected genes are reported. Data represent mean ± SD of three technical repeats from three biological replicates.

To dissect the genetic effects produced by SiO_2_NPs, we compared the global gene expression profiles of *Hydra* treated with sub-lethal dose (25 nM, 24 h) of nanoparticles versus control *Hydra*. Groups of 25 *Hydra* showing the inverted-cone morphology (score 5) or untreated were processed for RNA extraction and deep sequencing-based transcriptome analysis by RNAseq technology (Illumina). To calculate transcript coverage and differential gene expression, we employed, as reference, the annotated *Hydra* transcriptome deposited at the ENA. Ninety-three percent of the 100 base pairs sequencing reads from SiO_2_NPs treated *Hydra* were found to be uniquely mapped on the reference set (see “Materials and Methods” for details). Comparative analysis of transcripts indicated 45 DE genes (DE) across treatments. Interestingly, the relatively small number of DE transcripts may denote a low genotoxicity of SiO_2_NPs. Among these genes, 29 transcripts were upregulated (2-fold to 25-fold) and 16 were down regulated (−2.2-fold to −5-fold). All DE transcripts, their related annotations and increasing or decreasing fold changes are reported in Tables 3 and 4, respectively. Some of the modulated transcripts were annotated as related to cellular stress responses suggesting that the treatment effects can be transduced to the nucleus and influence the gene expression. In order to validate the RNAseq results, we selected three DE genes to be further analyzed by qRT-PCR on animals subjected to the same treatment. Figure 8 shows the relative expression level of *ppod, apx*-like, and *hsp16.2* genes obtained by qRT-PCR experiments confirming the expression changes detected by RNAseq (Tables 3 and 4). Interestingly, among the validated transcripts, there is the *ppod* gene, encoding for the *Hydra* PPOD: a secreted protein of cuticle (Bottger et al., 2012). This is the first functional evidence for downregulation of the *ppod* gene in response to external stimulus and its potential involvement in morphological and behavioral stress-related changes.

**Table 3:** **The table reports the RNAseq results for DE genes with fold changes above 2 (upregulated genes)**.

Sequence name	Annotation	Fold change
ENA|HAAC01032462|HAAC01032462.1	Pg1 homology to homo sapiens	25.26
ENA|HAAC01025529|HAAC01025529.1	Protein	14.03
ENA|HAAC01028000|HAAC01028000.1	Orf16–lacz fusion protein	14.03
ENA|HAAC01044255|HAAC01044255.1	60s ribosomal protein l18	12.21
ENA|HAAC01033821|HAAC01033821.1	Conserved protein	6.40
ENA|HAAC01033389|HAAC01033389.1	Glycosyl transferase (glycosyl transferase family 2)	6.31
ENA|HAAC01044227|HAAC01044227.1	Elongation factor 1-gamma	5.19
ENA|HAAC01028897|HAAC01028897.1	Pg1 homology to homo sapiens	5.05
ENA|HAAC01042994|HAAC01042994.1	Guanine nucleotide-binding protein subunit beta-2-like 1	4.75
ENA|HAAC01042461|HAAC01042461.1	Aminopeptidase n	4.42
ENA|HAAC01039586|HAAC01039586.1	Conserved protein	4.35
ENA|HAAC01018639|HAAC01018639.1	Conserved protein	4.33
ENA|HAAC01031633|HAAC01031633.1	Ascorbate peroxidase-like	4.04
ENA|HAAC01030032|HAAC01030032.1	Conserved protein	4.03
ENA|HAAC01029942|HAAC01029942.1	Ribosomal protein l44	3.95
ENA|HAAC01044395|HAAC01044395.1	40s ribosomal protein s4	3.72
ENA|HAAC01044240|HAAC01044240.1	Predicted: uncharacterized protein LOC100207688	3.71
ENA|HAAC01044744|HAAC01044744.1	Arminin-like peptide 27077	3.70
ENA|HAAC01043749|HAAC01043749.1	Predicted: uncharacterized protein LOC100211198	3.65
ENA|HAAC01044188|HAAC01044188.1	Predicted: uncharacterized protein LOC100207688	3.60
ENA|HAAC01032676|HAAC01032676.1	Predicted: uncharacterized protein LOC100200837	3.56
ENA|HAAC01017557|HAAC01017557.1	Ascorbate peroxidase-like	3.19
ENA|HAAC01043858|HAAC01043858.1	Lysosome-associated membrane glycoprotein 2-like	3.11
ENA|HAAC01000451|HAAC01000451.1	Cytoplasmic polyadenylation element-binding prot 1 isoform x3	3.07
ENA|HAAC01043989|HAAC01043989.1	Predicted: uncharacterized protein LOC100207688	3.00
ENA|HAAC01044084|HAAC01044084.1	Predicted: uncharacterized protein LOC100203182	2.91
ENA|HAAC01022047|HAAC01022047.1	Ubiquitin-conjugating enzyme e2 g2	2.90
ENA|HAAC01045088|HAAC01045088.1	tpa_exp: minicollagen 7	2.85
ENA|HAAC01012792|HAAC01012792.1	60 kDa lysophospholipase	2.34

*The sequence names are referred to the deposited *Hydra* transcriptome at European Nucleotide Archive (ENA) with relative annotations and fold changes*.

**Table 4:** **The table reports the RNAseq results for DE genes with fold changes below 0.5 (downregulated genes)**.

Sequence Name	Annotation	Fold change
ENA|HAAC01029048|HAAC01029048.1	Rhamnose-binding lectin-like	0.44
ENA|HAAC01040476|HAAC01040476.1	Heat shock cognate 71 kDa protein isoform x2	0.41
ENA|HAAC01005216|HAAC01005216.1	Gtp-binding protein rhes-like	0.40
ENA|HAAC01044402|HAAC01044402.1	Ppod1 peroxidase	0.39
ENA|HAAC01043441|HAAC01043441.1	Heat shock cognate 71 kDa protein	0.38
ENA|HAAC01003999|HAAC01003999.1	Predicted: uncharacterized protein LOC101235185	0.37
ENA|HAAC01019715|HAAC01019715.1	Hsp16.2	0.34
ENA|HAAC01029657|HAAC01029657.1	Heat shock cognate 71 kDa protein	0.33
ENA|HAAC01043390|HAAC01043390.1	Ppod2	0.30
ENA|HAAC01031772|HAAC01031772.1	DNAj homolog subfamily b member 4	0.30
ENA|HAAC01018572|HAAC01018572.1	Hsp16.2	0.28
ENA|HAAC01009426|HAAC01009426.1	Hypothetical protein DAPPUDRAFT_322288	0.27
ENA|HAAC01024155|HAAC01024155.1	Bag family molecular chaperone regulator 3-like	0.27
ENA|HAAC01031161|HAAC01031161.1	Family 2 glycosyl transferase	0.25
ENA|HAAC01028344|HAAC01028344.1	Predicted: uncharacterized protein LOC100203540	0.23
ENA|HAAC01027133|HAAC01027133.1	Hsp-16.2	0.18

*The sequence names are referred to the deposited *Hydra* transcriptome at European Nucleotide Archive (ENA) with relative annotations and fold changes*.

## Discussion

Previous results (Malvindi et al., 2012) suggest that SiO_2_NPs internalize into cultured cells and are biocompatible when used at low concentration (up to 2.5 nM). Here, we translated the study of SiO_2_NPs effects on an animal model analyzing the effects from 10 nM onward: a range reasonably higher in respect to *in vitro* experiments due to the required mode of NP administration, i.e., in addition to the animal culture medium. Dose response experiments indicated low toxicity up to 10 nM SiO_2_NPs; additionally, optical microscopy observation and ICP, indicated the ability of SiO_2_NPs to be internalized by ectodermal cells. Taken together, these results confirm the data produced *in vitro* (Malvindi et al., 2012). On the other hand, in the same experimental conditions (10 nM SiO_2_NPs), although in the absence of morphophysiological traits of toxicity, an increase in the apoptotic cell number and an impairment of the ability to regenerate amputated body region was observed. Nowak et al. (2014), in a recent paper, described as the internalization of SiO_2_NPs may activate survival mechanisms in cultured cells and how this reaction was mediated by augmented autophagy and not by apoptosis. These results suggest, for accurate toxicological investigations, that SiO_2_NPs can elicit effects even at sub lethal doses. Above 10 nM SiO_2_NPs, we observed worsening morphophysiological traits. Previous works, to measure the impact of toxicants on *Hydra*, used a score key based on progressive morphological alteration of animal anatomy that was employed to test water pollution, estrogens, and metal contamination (Wilby and Tesh, 1990; Ambrosone et al., 2012). Taking into account the new morphophysiological traits produced by SiO_2_NPs on *Hydra*, here we defined a new score key. The scores formed the raw data to obtain median values and LT50 and consequently delineate the lethal and sub-lethal incubation and concentration ranges. Through this analysis, we defined two frames of SiO_2_NPs toxicity in *Hydra*. At intermediate concentration (25 nM), sub lethal reversible effects were recorded as anatomical changes, impairment of feeding behavior, alterations of specialized cell morphology and organization, and global gene expression modulation. We set the inverted-cone (similar to a party hat) morphology as symptom of SiO_2_NPs sub-lethal toxicity. Remarkably, this condition resulted independent from the incubation time suggesting a low bioaccumulation of amorphous silica nanoparticles in *in vivo* experiments as described by other groups in *in vitro* studies (Gazzano et al., 2012). At higher concentration (35 nM) and after prolonged exposure (44 h), we observed irreversible effects and lethality. Interestingly, the sub-lethal toxicity traits do not show a linear correlation with the amount of internalized SiO_2_NPs. The discrepancy between morphophysiological effects and limited amount of SiO_2_NPs in *Hydra* tissues can be explained assuming that the nanoparticles interact with outer animal surface. Recent evidences report that the SiO_2_NPs *in vitro* toxicity is mediated by adsorption of nanoparticles to extracellular components as serum proteins (Napierska et al., 2010; Zhang et al., 2012). It has been demonstrated that the enormous nanoparticles surface area and their concentration of silanol groups enhance the binding of large quantities of proteins and other biomolecules (Izak-Nau et al., 2013). Here we suggest that, this interaction in the case of *Hydra*, takes place with the external cuticle, a thick multilayered extracellular matrix that selectively protect *Hydra* tissues from external agents. The subtraction of cuticle components by SiO_2_NPs, triggers the impairment of the homeostasis of *Hydra*/environment interface producing the inverted-cone condition described into results, this alters the equilibrium with external environment and influences basic functions as, for example, the effectiveness of feeding behavior and the hydrostatic pressure that mediate the spontaneous *Hydra* gastric region contractions. Nonetheless, the rather long LT50 could mean that, for about 38 h of continuous exposure to 35 nM SiO_2_NPs, *Hydra* can renew the cuticle components replenishing the cuticle components necessary to prevent the direct access of SiO_2_NPs to ectodermal epithelial cells. Anyway, prolonged exposition to high SiO_2_NPs concentration (44 h, SiO_2_NPs above 35 nM), acts irreversibly on *Hydra* tissue with extensive cell lysis and subsequent reduction of polyps size and irregular shape. Finally, as already described in cell culture experiments (Costantini et al., 2011) and paralleling the effects at the base of the hemolysis activity (Zhang et al., 2012), once extinguished the homeostatic potential of extracellular matrix machine, the direct contact of SiO_2_NPs with ectodermal epithelial cells, induces the rupture of the plasma membrane resulting in massive cell loss and animal death.

Notwithstanding the widespread use of SiO_2_NPs in cosmetics, medicine, and foods, a few experimental data are available to date on the effects of SiO_2_NPs on gene expression modulation (Nowak et al., 2014). We were interested to find at what extent the observed reversible effects elicited by SiO_2_NPs in *Hydra* had an echo on global gene expression levels. Transcriptome analysis showed a moderate effect of SiO_2_NPs treatment on gene expression confirming the overall low toxicity of SiO_2_NPs. Remarkably, among the genes whose expression was modulated by SiO_2_NPs treatment, we found out the *ppod* gene encoding the *Hydra* protein PPOD a well-known cuticle component (Bottger et al., 2012). Both RNAseq and qRT-PCR showed that the *ppod* gene transcript level decreases after SiO_2_NPs treatment. This secreted protein, shows hemagglutination activity and is supposed to be involved in organizing the cuticle glycosaminoglycans. This is a first evidence of *ppod* gene expression modulation in response to an external stimulus, and suggests a molecular counterpart of the observed morphophysiological modifications. Further studies will also clarify the mechanism underlying the down regulation of the ppod gene transcript as, for example, a putative negative feedback to the stress induced by SiO_2_NPs treatment. Since the transcriptome analysis was carried out after 24 h of treatments, the gene expression modulation can be considered a hallmark of early response to the toxic stimulus that form a first example of genes involved in dynamic regulation of *Hydra* with the aquatic environments in mild toxic condition (Nowak et al., 2014). To verify the robustness of RNAseq results, we further validated the expression level changes of the *HSP20* and *ascorbate peroxidase-like*. These genes with other DE genes as, for example, the *guanine nucleotide-binding protein subunit beta-2-like1* and *lysosome-associated membrane glycoprotein-2-like* genes are connected with cellular response to chemical stimuli, toxicants, and apoptosis, reinforcing the hypotheses of SiO_2_NPs-induced cellular stress. Finally, in our RNA-seq analysis, a sizeable number of genes remain unknown, providing a valid source of functional information to be further investigated. Future experiments and data analysis will be necessary to place these genes in functional pathways as molecular correlates of the morphophysiological traits reported in this work.

Our results describe new biological impacts from nanoscale materials and the data provide useful evidences for the development of novel atlas for the prediction of effects of nanomaterial interaction with living organisms. These results suggest the necessity to screen multiple experimental models and to apply a wide range of tests to better describe the toxicity endpoints of any new nanomaterial.

## Conflict of Interest Statement

The authors declare that the research was conducted in the absence of any commercial or financial relationships that could be construed as a potential conflict of interest.

## Supplementary Material

The Supplementary Material for this article can be found online at http://www.frontiersin.org/Journal/10.3389/fbioe.2014.00037/abstract

## Footnotes

^1^http://www.metazome.net/

^2^http://www.ebi.ac.uk/ena/

^3^http://frodo.wi.mit.edu/primer3/

^4^http://www.bioinformatics.babraham.ac.uk/projects/fastqc

^5^http://bio-bwa.sourceforge.net

^6^http://www.ncbi.nlm.nih.gov

## References

[B1] Ambrosone A. Mattera L. Marchesano V. Quarta A. Susha A. S. Tino A. (2012). Mechanisms underlying toxicity induced by CdTe quantum dots determined in an invertebrate model organism. Biomaterials 33, 1991–2000 10.1016/j.biomaterials.2011.11.041 22169823

[B2] Ambrosone A. Tortiglione C. (2013). Methodological approaches for nanotoxicology using Cnidarian models. Toxicol. Mech. Methods 23, 207–216 10.3109/15376516.2012.747117 23193991

[B3] Anders S. Huber W. (2010). Differential expression analysis for sequence count data. Genome Biol. 11, R106 10.1186/gb-2010-11-10-r106 20979621 PMC3218662

[B4] Ashburner M. Ball C. A. Blake J. A. Botstein D. Butler H. Cherry J. M. (2000). Gene ontology: tool for the unification of biology. The Gene Ontology Consortium. Nat. Genet. 25, 25–29 10.1038/75556 10802651 PMC3037419

[B5] Bode H. (2011). Axis formation in hydra. Annu. Rev. Genet. 45, 105–117 10.1146/annurev-genet-102209-163540 21819240

[B6] Bosch T. C. David C. N. (1984). Growth regulation in Hydra: relationship between epithelial cell cycle length and growth rate. Dev. Biol. 104, 161–171 10.1016/0012-1606(84)90045-9 6734933

[B7] Bottger A. Doxey A. C. Hess M. W. Pfaller K. Salvenmoser W. Deutzmann R. (2012). Horizontal gene transfer contributed to the evolution of extracellular surface structures: the freshwater polyp hydra is covered by a complex fibrous cuticle containing glycosaminoglycans and proteins of the PPOD and SWT (sweet tooth) families. PLoS ONE 7:e52278 10.1371/journal.pone.0052278 23300632 PMC3531485

[B8] Costantini L. M. Gilberti R. M. Knecht D. A. (2011). The phagocytosis and toxicity of amorphous silica. PLoS ONE 6:e14647 10.1371/journal.pone.0014647 21311600 PMC3032735

[B9] David C. (1973). A quantitative method for maceration of hydra tissue. W. Roux’ Archiv. f. Entwicklungsmech. 171, 259–268 10.1007/BF00577724 28304607

[B10] Fujisawa T. (1992). Homeostatic recovery of interstitial cell populations in Hydra. Dev. Biol. 150, 185–192 10.1016/0012-1606(92)90017-B 1537433

[B11] Galliot B. Quiquand M. Ghila L. De Rosa R. Miljkovic-Licina M. Chera S. (2009). Origins of neurogenesis, a Cnidarian view. Dev. Biol. 332, 2–24 10.1016/j.ydbio.2009.05.563 19465018

[B12] Gazzano E. Ghiazza M. Polimeni M. Bolis V. Fenoglio I. Attanasio A. (2012). Physicochemical determinants in the cellular responses to nanostructured amorphous silicas. Toxicol. Sci. 128, 158–170 10.1093/toxsci/kfs128 22491428

[B13] Gene Ontology C. (2008). The gene ontology project in 2008. Nucleic Acids Res. 36, D440–D444 10.1093/nar/gkm883 17984083 PMC2238979

[B14] Gotz S. Garcia-Gomez J. M. Terol J. Williams T. D. Nagaraj S. H. Nueda M. J. (2008). High-throughput functional annotation and data mining with the Blast2GO suite. Nucleic Acids Res. 36, 3420–3435 10.1093/nar/gkn176 18445632 PMC2425479

[B15] Guarnieri D. Malvindi M. A. Belli V. Pompa P. P. Netti P. (2014). Effect of silica nanoparticles with variable size and surface functionalization on human endothelial cell viability and angiogenic activity. J. Nanoparticle Res. 16, 10.1007/s11051-013-2229-6

[B16] Hamilton M. A. Russo R. C. Thurston R. V. (1977). Trimmed Spearman-Karber method for estimating median lethal concentrations in toxicity bioassays. Environ. Sci. Technol. 11, 714–719 10.1021/es60130a004

[B17] Hobmayer E. Holstein T. W. David C. N. (1990). Tentacle morphogenesis in hydra. 2. Formation of a complex between a sensory nerve-cell and a battery cell. Development 109, 897–904

[B18] Izak-Nau E. Voetz M. Eiden S. Duschl A. Puntes V. F. (2013). Altered characteristics of silica nanoparticles in bovine and human serum: the importance of nanomaterial characterization prior to its toxicological evaluation. Part. Fibre Toxicol. 10, 10.1186/1743-8977-10-56 24206572 PMC3829099

[B19] Karntanut W. Pascoe D. (2002). The toxicity of copper, cadmium and zinc to four different Hydra (Cnidaria: Hydrozoa). Chemosphere 47, 1059–1064 10.1016/S0045-6535(02)00050-4 12137038

[B20] Lenhoff H. M. (1961). Activation of the feeding reflex in Hydra littoralis. I. Role played by reduced glutathione and quantitative assay of the feeding reflex. J. Gen. Physiol. 45, 331–344 10.1085/jgp.45.2.331 14463986 PMC2195168

[B21] Li H. Durbin R. (2009). Fast and accurate short read alignment with Burrows-Wheeler transform. Bioinformatics 25, 1754–1760 10.1093/bioinformatics/btp324 19451168 PMC2705234

[B22] Livak K. J. Schmittgen T. D. (2001). Analysis of relative gene expression data using real-time quantitative PCR and the 2(T)(-Delta Delta C) method. Methods 25, 402–408 10.1006/meth.2001.1262 11846609

[B23] Loomis W. F. Lenhoff H. M. (1956). Growth and sexual differentiation of hydra in mass culture. J. Exp. Zool. 132, 555–573 10.1002/jez.1401320309

[B24] Malvindi M. A. Brunetti V. Vecchio G. Galeone A. Cingolani R. Pompa P. P. (2012). SiO2 nanoparticles biocompatibility and their potential for gene delivery and silencing. Nanoscale 4, 486–495 10.1039/c1nr11269d 22095171

[B25] Malvindi M. A. Carbone L. Quarta A. Tino A. Manna L. Pellegrino T. (2008). Rod-shaped nanocrystals elicit neuronal activity in vivo. Small 4, 1747–1755 10.1002/smll.200800413 18844306

[B26] Marchesano V. Hernandez Y. Salvenmoser W. Ambrosone A. Tino A. Hobmayer B. (2013). Imaging inward and outward trafficking of gold nanoparticles in whole animals. ACS Nano 7, 2431–2442 10.1021/nn305747e 23448235

[B27] Martinez D. E. Bridge D. (2012). Hydra, the everlasting embryo, confronts aging. Int. J. Dev. Biol. 56, 479–487 10.1387/ijdb.113461dm 22689361

[B28] Napierska D. Thomassen L. C. J. Lison D. Martens J. A. Hoet P. H. (2010). The nanosilica hazard: another variable entity. Part. Fibre Toxicol. 7, 39 10.1186/1743-8977-7-39 21126379 PMC3014868

[B29] Nowak J. S. Mehn D. Nativo P. García C. P. Gioria S. Ojea-Jiménez I. (2014). Silica nanoparticle uptake induces survival mechanism in A549 cells by the activation of autophagy but not apoptosis. Toxicol. Lett. 224, 84–92 10.1016/j.toxlet.2013.10.003 24140553

[B30] Pierobon P. Minei R. Porcu P. Sogliano C. Tino A. Marino G. (2001). Putative glycine receptors in hydra: a biochemical and behavioural study. Eur. J. Neurosci. 14, 1659–1666 10.1046/j.0953-816x.2001.01792.x 11860460

[B31] Pierobon P. Sogliano C. Minei R. Tino A. Porcu P. Marino G. (2004). Putative NMDA receptors in hydra: a biochemical and functional study. Eur. J. Neurosci. 20, 2598–2604 10.1111/j.1460-9568.2004.03759.x 15548203

[B32] Quinlan A. R. Hall I. M. (2010). BEDTools: a flexible suite of utilities for comparing genomic features. Bioinformatics 26, 841–842 10.1093/bioinformatics/btq033 20110278 PMC2832824

[B33] Ruggieri R. D. Pierobon P. Kass-Simon G. (2004). Pacemaker activity in hydra is modulated by glycine receptor ligands. Comp. Biochem. Physiol. A Mol. Integr. Physiol. 138, 193–202 10.1016/j.cbpb.2004.03.015 15275654

[B34] Thurm U. Brinkmann M. Golz R. Holtmann M. Oliver D. Sieger T. (2004). Mechanoreception and synaptic transmission of hydrozoan nematocytes. Hydrobiologia 530, 97–105 10.1007/978-1-4020-2762-8_12

[B35] Tortiglione C. Quarta A. Malvindi M. A. Tino A. Pellegrino T. (2009). Fluorescent nanocrystals reveal regulated portals of entry into and between the cells of Hydra. PLoS ONE 4:e7698 10.1371/journal.pone.0007698 19888325 PMC2765617

[B36] Tortiglione C. Quarta A. Tino A. Manna L. Cingolani R. Pellegrino T. (2007). Synthesis and biological assay of GSH functionalized fluorescent quantum dots for staining Hydra vulgaris. Bioconjug. Chem. 18, 829–835 10.1021/bc060355t 17441682

[B37] Wenger Y. Galliot B. (2013). RNAseq versus genome-predicted transcriptomes: a large population of novel transcripts identified in an Illumina-454 Hydra transcriptome. BMC Genomics 14, 10.1186/1471-2164-14-204 23530871 PMC3764976

[B38] Westfall J. A. Kinnamon J. C. (1984). Perioral synaptic connections and their possible role in the feeding behavior of Hydra. Tissue Cell 16, 355–365 10.1016/0040-8166(84)90055-7 6464003

[B39] Wilby O. K. Tesh J. M. (1990). The Hydra assay as an early screen for teratogenic potential. Toxicol. In vitro 4, 582–583 10.1016/0887-2333(90)90119-E 20702233

[B40] Zhang H. Y. Dunphy D. R. Jiang X. M. Meng H. Sun B. B. Tarn D. (2012). Processing pathway dependence of amorphous silica nanoparticle toxicity: colloidal vs pyrolytic. J. Am. Chem. Soc. 134, 15790–15804 10.1021/ja304907c 22924492 PMC3505689

