# Supplementary material for: Impact of Amorphous SiO2 Nanoparticles on a Living Organism: Morphological, Behavioral, and Molecular Biology Implications
Source: Front Bioeng Biotechnol. 2014 Sep 29;2:37. doi: 10.3389/fbioe.2014.00037 (PMC4179610; doi:10.3389/fbioe.2014.00037)
Supplement: Supplementary file 5 [file Data_Sheet1.DOCX]

Electronic Supplementary Material

Impact of Amorphous Silica Nanoparticles on a Living Organism: Morphological, Behavioural and Molecular Cell Biology Implications.

Alfredo Ambrosone^1^, Maria Rosaria Scotto Di Vettimo^1^, Maria Ada Malvindi^2^, Modi Roopin^3^, Oren Levy^3^, Valentina Marchesano^1^, Pier Paolo Pompa^2^, Claudia Tortiglione^1^, Angela Tino^1^(🖂).

^1^Istituto di Cibernetica “Eduardo Caianiello”, Consiglio Nazionale delle Ricerche, Via Campi Flegrei, 34 Pozzuoli NA 80078. Italy

^2^ Center for Biomolecular Nanotechnologies@UNILE, Istituto Italiano di Tecnologia, Arnesano, Italy

^3^ Department of Marine, Biology Charney School for Marine Sciences, The University of Haifa, Israel


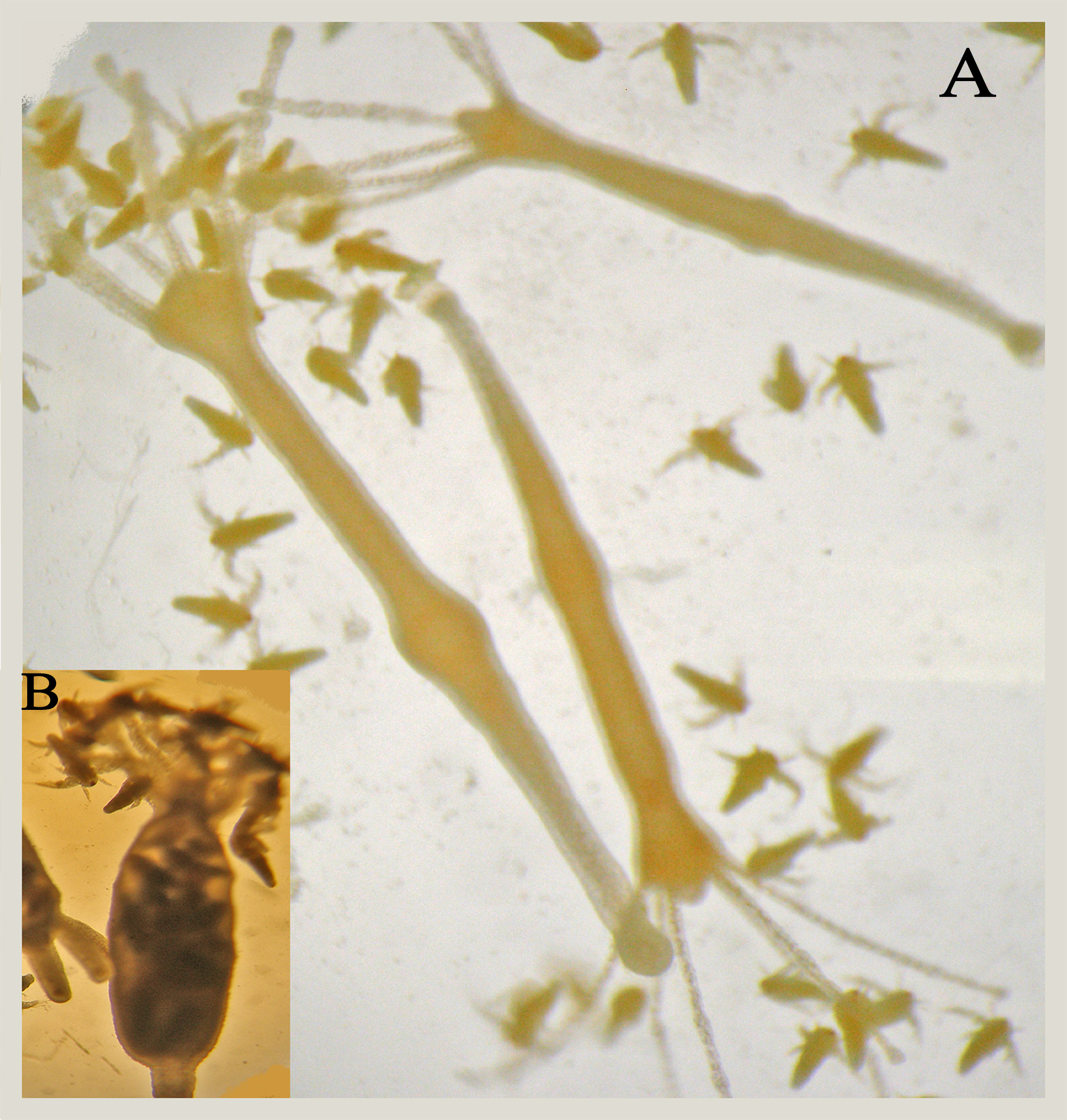


Figure S1 Supplemental Figure1. Feeding impairment of treated *Hydra* (a); Normal *Hydra* after feeding showing the gastric region fulfilled with *artemia salina* (b).

**Table S1**. List of genes whose expression was analyzed by qRT-PCR; the GenBank genetic sequence database I.D., and the corresponding specific sequences are reported.

| protein | accession number | Forward primer | Reverse primer |
| --- | --- | --- | --- |
| PPOD2 | AY034096 | GCC AAC AAA GAC CAG GCT AC | AGA ACA TCG CGG TTA GCT GT |
| APX-like | XM_002158799 | GGC ATT GAA GGA ACC GAT AA | CAA GCG TGT GTG CTC CTA AA |
| HSP20 | XM_004212174 | GGT GAA ACA AAA GCG GAA AG | TCA TCT GGC AAA GCT ACG TG |

## Supplementary Material legends

Supplemental Figure1. Feeding impairment of treated *Hydra* (a); Normal *Hydra* after feeding showing the gastric region fulfilled with *artemia salina* (b).

Table S1. List of genes whose expression was analyzed by qRT-PCR; the GenBank genetic sequence database I.D., and the corresponding specific sequences are reported

**Movie M1.** Normal Hydra feeding behavior: untreated *Hydra* challenged with living *Artemia nauplii* react seizing the preys and opening the mouths.

**Movie M2**. GSH response in untreated Hydra: after GSH administration Hydra mimic the feeding response

**Movie M3.** Feeding behavior impairment in treated Hydra. Treated Hydra are able to catch the preys but in absence of gastric contractions, do not open the mouth

**Movie M4.** GSH response impairment in treated Hydra. Treated Hydra starts the tentacles writhing but is unable to open the mouth.
